# Regional pressure and temperature variations across the injured human brain: comparisons between paired intraparenchymal and ventricular measurements

**DOI:** 10.1186/s13054-015-0982-x

**Published:** 2015-06-23

**Authors:** Charmaine Childs, Liang Shen

**Affiliations:** Centre for Health and Social Care Research, Sheffield Hallam University, Montgomery House, 32 Collegiate Crescent, Sheffield, S102BP UK; Department of Biostatistics, Deans Office, National University of Singapore, Level 6, Kent Ridge Wing, National University Hospital, Singapore, 119074 Singapore

## Abstract

**Introduction:**

Intraparenchymal, multimodality sensors are commonly used in the management of patients with severe traumatic brain injury (TBI). The ‘gold standard’, based on accuracy, reliability and cost for intracranial pressure (ICP) monitoring is within the cerebral ventricle (external strain gauge). There are no standards yet for intracerebral temperature monitoring and little is known of temperature differences between brain tissue and ventricle. The aim of the study therefore was to determine pressure and temperature differences at intraparenchymal and ventricular sites during five days of continuous neuromonitoring.

**Methods:**

Patients with severe TBI requiring emergency surgery. Inclusion criteria: patients who required ICP monitoring were eligible for recruitment. Two intracerebral probe types were used: a) intraventricular, dual parameter sensor (measuring pressure, temperature) with inbuilt catheter for CSF drainage: b) multiparameter intraparenchymal sensor measuring pressure, temperature and oxygen partial pressure. All sensors were inserted during surgery and under aseptic conditions.

**Results:**

Seventeen patients, 12 undergoing neurosurgery (decompressive craniectomy *n* = 8, craniotomy *n* = 4) aged 21–78 years were studied. Agreement of measures for 9540 brain tissue-ventricular temperature ‘pairs’ and 10,291 brain tissue-ventricular pressure ‘pairs’ were determined using mixed model to compare mean temperature and pressure for longitudinal data.

There was no significant overall difference for mean temperature (*p* = 0.92) or mean pressure readings (*p* = 0.379) between tissue and ventricular sites. With 95.8 % of paired temperature readings within 2SD (−0.4 to 0.4 °C) differences in temperature between brain tissue and ventricle were clinically insignificant. For pressure, 93.5 % of readings pairs fell within the 2SD range (−9.4756 to 7.8112 mmHg). However, for individual patients, agreement for mean tissue-ventricular pressure differences was poor on occasions.

**Conclusions:**

There is good overall agreement between paired temperature measurements obtained from deep white matter and brain ventricle in patients with and without early neurosurgery. For paired ICP measurements, 93.5 % of readings were within 2SD of mean difference. Whilst the majority of paired readings were comparable (within 10 mmHg) clinically relevant tissue-ventricular dissociations were noted. Further work is required to unravel the events responsible for short intervals of pressure dissociation before tissue pressure readings can be definitively accepted as a reliable surrogate for ventricular pressure.

## Introduction

Intracranial pressure (ICP) monitoring continues to form the internationally agreed parameter used to identify secondary cerebral deterioration in patients with a severe (Glasgow Coma Scale (GCS) 3–8) head injury [1]. This widely adopted measurement recommendation’s aim is to keep ICP below 20 mmHg, but whilst supported in the clinical setting it had not been confirmed by clinical ‘testing’ until the recent publication by Chestnut et al. [2]. Whilst the findings of this multicentre randomized controlled trial reveal no greater outcome benefit of protocol-guided (intraparenchymal) ICP monitoring over computed tomography (CT) imaging and clinical examination at the 3-month and 6-month post-traumatic brain injury (TBI) time point, it must not yet be the time to disinvest in the potential advantages of ICP measurement per se. Without physiological measurement, we risk a retrograde step; rather, we should aim to understand the vagaries of clinical measurement and to endeavour to evaluate the accuracy, reliability and stability of our measuring devices [3, 4]; a timely consideration in view of the increasing number of sensors now marketed for ICP measurement as well as the probes emerging for tissue and ventricular temperature (and oxygen content).

The long-held ‘gold standard’ technique for ICP measurement is via a catheter placed in the lateral ventricle, typically via a small right frontal burr hole [5]. Pressure readings are obtained either via the cerebrospinal fluid (CSF)-filled catheter attached to an external transducer [6] or, as more recently available, by microsensors implanted within the tip of the sensor [7]. Alternatively and commonly, ICP readings are obtained via microsensor systems implanted in the cerebral parenchyma [8].

In ‘next-generation’ systems, ICP can be measured in conjunction with temperature, tissue oxygen [9] and chemistry; and as the era of cerebral multimodality monitoring progresses, opportunities arise to be fully cognizant of the accuracy and reliability of the sensors and the instruments [3]. In addition, appreciation of the nature of regional variations (pressure and temperature, for example) serves to improve our certainty of ‘true’ intracranial measurement. This is particularly important if non-invasive systems [10] are to be validated against invasive systems. Here, the key issue is in the extent of site-specific pressure and temperature gradients.

### Objective

In view of the potential for distortion and effacement of the ventricular system owing to intracerebral unilateral or bilateral lesions, insertion of sensors into the lateral ventricle is often extremely difficult. Intraparenchymal sensors provide an alternative solution. The objective of this study was to determine the extent of correspondence at intraparenchymal (tissue) and ventricular sites for pressure and temperature in patients with and without emergency neurosurgery for severe TBI.

## Methods

### Study design

A prospective, observational study was carried out in a cohort of patients with severe TBI.

### Participants

Patients with severe TBI and with a range of GCS on admission (Table 1) who required emergency surgery for their brain injury and who, postoperatively and following standard care, required CSF drainage via an extraventricular drain together with ICP monitoring were eligible for recruitment to the study. Study data were acquired during 5 days from insertion of the sensors in the setting of neurocritical care.

**Table 1:** Patient demographics, injury and severity, and neuromonitoring (site and depth) with 30-day and 3-month patient outcome

ID	Race	Injury	AIS	ISS	Isolated TBI	Admission GCS^a^	Hours after injury at start of monitoring	Sensor site depth^b^ (cm)	Outcome (days, months)	
									30 days	3 months
1	Chinese	Fall	5	25	Yes	14/15 and 8/15	37	V-right T-right 2.4	2	2
2	Chinese	Fall	5	25	Yes	8/15	17	V-right T-left 4.7	4	4
3	Chinese	RTC	5	35	No	3/15	44^c^	V-right T-right 4.2	2	4
4	Chinese	Fall	5	25	Yes	7/15	8	V-left T-left 2.3	2	2
5	Burmese	RTC	5	29	No	7/15	18	V-right T-right 2.0	4	4
6	Chinese	Fall	4	16	Yes	14/15	7	V-left T-right 3.7	4	4
7	Malay	RTC	5	33	No	14/15 and 8/15	14	V-left T-left 4.0	4	4
8	Chinese	Fall	5	25	Yes	15/15 and 8/15	10	V-left T-left 2.0	1	1
9	Chinese	Fall	4	35	No	9/15	47	V-right T-right 2.6	3	3
10	Indian	RTC	4	16	Yes	6/15	8	V-right T-left 3.1	1	1
11	Indian	Fall	5	25	Yes	10/15	7	V-left T-left 3.2	1	1
12	Indian	Fall	5	25	Yes	6/15	7	V-left T-right 2.6	1	1
13	Malay	RTC	4	16	Yes	3/15	60^d^	V-right T-right 3.2	3	4
14	Chinese	RTC	4	16	Yes	14/15	3	V-right T-left 4.2	3	4
15	Malay	RTC	5	25	Yes	8/15	10	V-right T-right 3.0	3	3
16	Chinese	RTC	4	16	Yes	4/15	9	V-left T-left 5.4	1	1
17	Chinese	Fall	5	25	Yes	8/15	8	V-left T-left 5.0	1	1

^a^Where two values are given, this shows GCS deterioration during the admission period (where documented)

^b^Sensor sites: *V* ventricle (Neurovent-Temp probe; Raumedic™, Munchberg, Germany) right or left; *T* tissue and depth (cm)

^c^Sensor inserted 44 hours after TBI (delayed due to neurosurgery and orthopaedic surgery)

^d^Sensor insertion delayed due to coagulopathy

*AIS* Abbreviated Injury Scale, *GCS* Glasgow Coma Scale, *ISS* Injury Severity Score, *RTC* road traffic crash, *TBI* traumatic brain injury

### Intracerebral monitoring

Two sensor types were used, both manufactured by Raumedic™ (Munchberg, Germany). The Neurovent-Temp-IFD-S-C is a dual-parameter probe designed for insertion into the lateral cerebral ventricle. The catheter lumen allows for drainage of CSF. The probe also houses two microsensors for measurement of pressure (mmHg) and temperature (°C). A second sensor (Neurovent-PTO) measuring pressure, temperature and oxygen partial pressure (mmHg; oxygen data not shown) was inserted into brain tissue white matter. In this study, the target site was the right (non-eloquent) uninjured frontal lobe but at times, owing to tissue damage, the contralateral lobe was used. Whenever possible, the sensor tip position was confirmed by radiological (CT) examination for measurement depth (cm). All sensors were inserted during surgery and under aseptic conditions.

### Clinical monitoring

After insertion of the sensor in the operating room and before transport of the patient to the ICU, the sensors were checked to establish correct functioning and readings. On arrival in the ICU the Neurovent-Temp dual-parameter probe, measuring ventricular temperature and pressure (and drainage of CSF), was connected, via a ‘plug and play’ system, to the bedside data acquisition system of the ICU (GE Solar 8000i; Buckinghamshire, UK) using proprietary cabling. Data from this system were stored in an Intellivue Clinical Information Portfolio (ICIP; Philips, Eindhoven, Netherlands) at sampling rates of 2 Hz.

Readings from the second, Neurovent-PTO multiparameter, sensor (measuring pressure, temperature and oxygen content) were obtained via a ‘stand-alone’ datalogger (MPR2 Log O; Raumedic™). The data logger was necessary owing to limited availability of ‘channels’ on the bedside monitoring system ‘rack’.

Data from the ICIP server and from the Raumedic™ datalogger were imported separately to the Microsoft Structured Language Query SQL server 2000. This allowed the organization of ‘raw data’ (typically at a sampling rate of 6 readings/minute) and processing to achieve values averaged over 1-minute intervals to ensure standardization between the time intervals of the bedside system and the data logger. Owing to the large volume of data values for each parameter, readings were subsequently averaged over 5-minute and 10-minute intervals. Here, data averaged over 10 minutes are presented for the purposes of statistical analysis for each parameter and each patient. Differences between intracerebral readings obtained via the intraparenchymal and ventricular sensor (pressure, temperature) are presented throughout as tissue minus ventricle readings.

### Injury assessment

The extent and severity of peripheral injury was assessed using the Injury Severity Scoring System, an internationally recognized, consensus-derived, trauma scale [11]. A numerical score is allocated to individual injuries by body region (six regions in total) on a 6-point ordinal scale (Abbreviated Injury Scale (AIS)) ranging from AIS 1 (minor) to AIS 6 (currently untreatable). The AIS for the head includes the cranium and brain. The total Injury Severity Score (ISS) was calculated as the sum of squares of the numerical AIS of the three highest scoring body regions.

### Follow-up and assessment of outcome

Follow-up of neurological outcome using the Glasgow Outcome Score (GOS) was undertaken at the 30-day and 3-month time points after injury. If patients remained as ‘in-patients’, GOS assessment was undertaken in the hospital (and with the patients’ nurse) if the patient had not regained capacity. At the 3-month time point, follow-up assessment was undertaken with either the patient himself/herself or with the patients’ carer.

### Statistical analysis

Agreement of measures of brain temperature and pressure between different regions was evaluated by Bland–Altman plot. A mixed model for longitudinal data was utilized to compare the mean temperature and pressure between brain tissue and cerebral ventricle sites. The predictors of the difference of brain temperature and pressure between these two regions were studied using a mixed model. A Bland–Altman plot was drawn using (IBM Singapore, Singapore SPSS v.20) and the mixed model was performed using (SAS Cary, NC USA v.9.0).

### Ethics

Ethics approval from the Domain Specific Review Board National Health Group, Singapore was obtained before the study commenced. Written informed consent was obtained from a relative, spouse or legally acceptable representative before neurosurgery.

## Results

### Patients

Seventeen patients (12 male) aged 21–78 years (median 47 years) and predominantly of Chinese race (*n* = 10) were recruited (Table 1). Cause of injury were falls from height (*n* = 9) and road traffic crashes (*n* = 8) resulting in an ISS of 16–38 (median 25). Isolated injury to the head occurred in 13 patients (Table 1) with an injury severity (AIS brain) of AIS 4 (*n* = 7) and AIS 5 (*n* = 10), commensurate with severe and critical injury respectively.

### Neuromonitoring

Monitoring, including vital signs, commenced at the end of neurosurgery, 3–60 hours (median 10 hours) after injury (Table 1). Late neuromonitoring (i.e. >36 hours after injury) occurred in four patients due to sudden neurological deterioration and need for intensive medical care. All patients had insertion of both intraparenchymal and intraventricular sensors. Twelve patients underwent decompressive craniectomy.

The tissue sensor tip was positioned in situ to an average depth of 3.1 cm (range 2.0–5.4 cm). In 10 patients the intraparenchymal sensor was positioned predominately in deep white matter of the right lobe at a depth of 2.0–5.4 cm (median 3.1 cm) and predominately in undamaged (‘normal’) tissue (Table 1). In three patients, sensors were positioned in tissue—identified radiologically (CT) after insertion as borderline ‘normal’. In one patient, the sensor was noted to be in an area of tissue ischaemia.

Study data were collected during the acute phase (first 5 days) but sensors remained in situ until removal on the advice of the attending neurosurgeon.

There were six early deaths (days 2–6 after TBI). At 30 days after injury there were 11 survivors. GOS at 3 months was unfavourable in four patients (GOS 2, *n* = 2; GOS 3, *n* = 2) and favourable in seven patients (GOS 4). None of the survivors had a GOS 5 at the follow-up assessment (Table 1).

### Regional temperature differences

In total 9540 brain tissue and cerebral ventricle temperature pairs were obtained for the 17 patients during the first 5 days after surgery or admission to the ICU (Table 2). There was no significant difference between mean brain tissue temperature and mean ventricular temperature (*p* = 0.918). The mean difference between tissue and ventricular temperature was 0.0133 °C. Mean brain tissue temperature was slightly (0.0051 °C) below mean ventricular temperature (95 % CI −0.10 to 0.11), showing neither statistically significant nor clinically significant differences between the two brain sites.

**Table 2:** Differences in temperature between brain sites (tissue minus ventricle) for 17 patients studied during the course of 5 days of multiparameter neuromonitoring

Differences in temperature between brain sites (tissue minus ventricle, °C)					
ID	Number	Range		Mean	SD
1	712	0.06	0.53	0.2543	0.05341
2	717	−0.20	0.27	−0.0800	0.05657
3	665	0.08	0.63	0.3469	0.08055
4	685	−0.41	0.46	0.0631	0.15677
5	687	−0.16	0.93	0.1487	0.14814
6	630	−0.11	0.35	0.0434	0.06761
7	99	0.17	0.34	0.2457	0.03145
8	679	−0.46	0.03	−0.1291	0.09426
9	715	−0.47	0.16	−0.1439	0.13280
10	651	0.01	0.26	0.1223	0.03605
11	841	−0.24	0.36	0.0150	0.04957
12	274	−1.96	−0.30	−0.4374	0.13260
13	302	−0.23	0.07	−0.0725	0.05741
14	694	−0.05	0.48	0.0915	0.03959
15	702	−0.39	−0.10	−0.2338	0.06450
16	173	−0.53	0.01	−0.1200	0.05569
17	314	−1.36	0.54	−0.2009	0.31931

Number of temperature measurement pairs, range of differences (lowest to highest), mean difference between sites and standard deviation (SD)

**Fig. 1:**
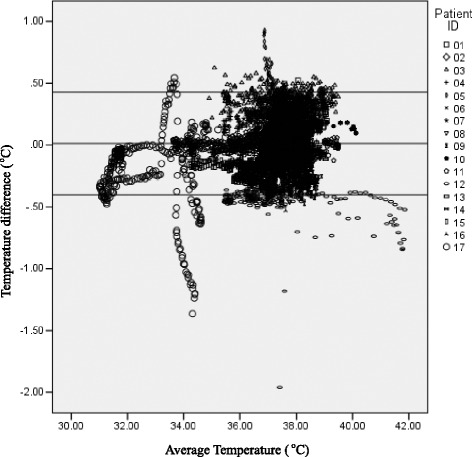
Temperature differences between brain sites (tissue minus ventricle) for 17 patients showing maximal differences; tissue temperature 1.96 °C below ventricular temperature for Patient 12 (GOS 1 at 30 days), and tissue 1.36 °C below ventricular temperature for Patient 17 (GOS 1 at 30 days). Mean difference for the group, 0.013 °C. For the majority of measurement pairs (95.8 %) the temperature of tissue and ventricular sites differ from each other, in either direction by 0.50 °C. For the group, differences in temperature between the two sites are not significant

Temperature agreement was evaluated (Fig. 1). In 95.8 % of temperature measurement pairs, differences between the sites fell within a two standard deviation (2SD) range (−0.40168 to 0.42828 °C). This indicates a broadly acceptable clinical agreement (0.4 °C) between intracerebral (tissue, ventricle) sites. However, at times differences between tissue and ventricular sites exceeded the upper and lower limits of agreement (Fig. 1) with maximal differences of −1.96 and 0.93 °C (Table 2). Temperature differences (tissue temperature below ventricular temperature) in excess of 2SD were observed only in non-survivors (Patients 12 and 17).

Furthermore, neither mean brain tissue temperature (*p* = 0.8995) nor mean ventricular temperature (*p* = 0.9783) were significantly different from core body (rectal) temperature. The mean difference between brain tissue temperature and core body (rectal) temperature was 0.018 °C (95 % CI −0.28 to 0.31 °C) and the mean difference between mean ventricular temperature and mean rectal temperature was −0.0041 (95 % CI −0.32 to 0.31 °C).

Exploring for predictors of temperature differences (tissue minus ventricular temperature) on outcome (favourable vs. unfavourable), there was a borderline effect (*p* = 0.0524); patients with outcome (GOS 2) had slightly greater differences (by 0.21 °C) compared with patients with GOS 3. There was no significant effect (*p* = 0.9544) of the mean difference between the sites for those patients undergoing neurosurgery versus those who received intracerebral monitoring (plus CSF drainage) only (Table 2). For all other parameters measured and explored (brain tissue partial pressure (Pb_ti_O_2_), peripheral capillary oxygen saturation (SpO_2_), mean arterial pressure (MAP, mmHg), heart rate (beats/minute), expired carbon dioxide (end tidal CO_2_)), each one contributed a statistically significant (*p* = 0.0001, *p* < 0.0001, *p* = 0.0516, *p* < 0.0001, *p* = 0.0001 respectively) effect on the mean difference in temperature between the two brain sites. However, the effect was indeed minor and clinically irrelevant (i.e. less than 0.1 °C).

### Regional pressure differences

**Fig. 2:**
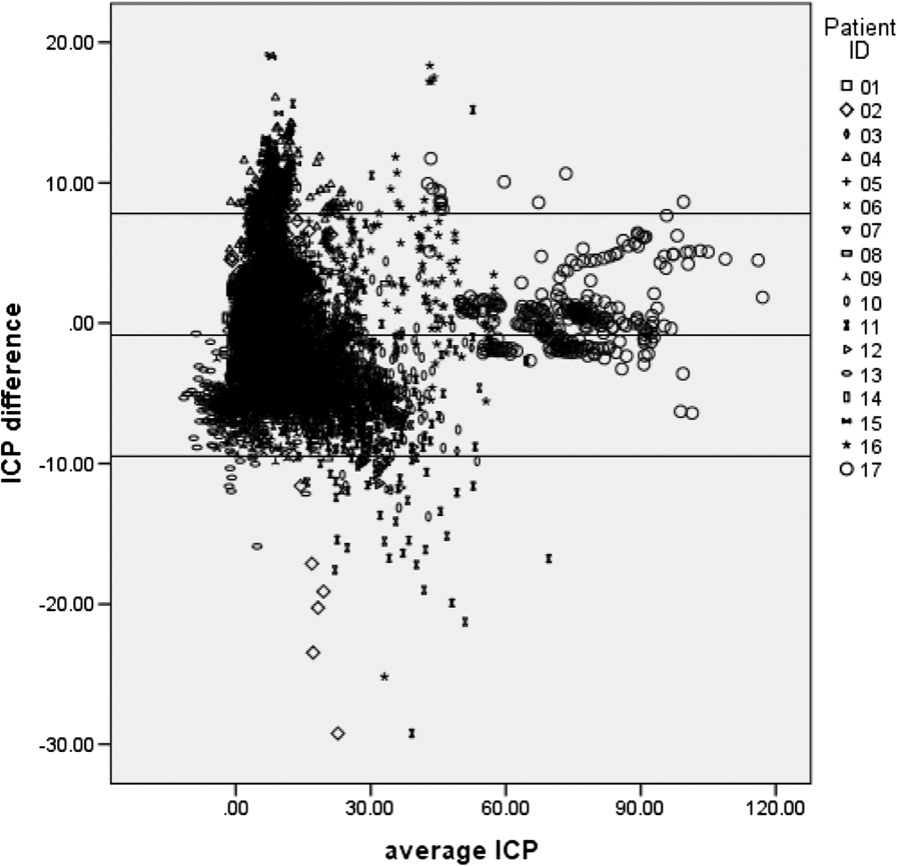
Pressure differences between brain sites (tissue minus ventricle) for 17 patients. For the majority of measurements (93.5 %), the difference in pressure (within 2SD of the mean) varied in either direction by close to 10 mmHg. For three patients (Patients 11, 2 and 16; GOS 1, 4 and 1 respectively) tissue pressure was frequently between 10 and 30 mmHg lower than ventricular pressure. For two patients (Patients 4 and 6; GOS 2 and 6 respectively) tissue pressure was frequently greater than ventricular pressure. For the group, the average difference between sites was minimal (−0.832 mmHg) indicating that tissue pressure, on average, was ‘minimally’ lower than ventricular pressure; a clinically insignificant (average) difference between tissue and ventricular sites. *ICP* intracranial pressure

For measurement of ICP 10,291 brain tissue–ventricular reading pairs were obtained. Overall, the mixed model showed no significant difference in mean pressure readings between brain tissue and ventricular readings (*p* = 0.379, mean difference = −0.80, 95 % CI −2.66 to 1.07), with 93.5 % of brain pressure readings pairs falling within the 2SD range (−9.4756 to 7.8112 mmHg) (Fig. 2).

However, for individual patients, agreement for mean tissue–ventricular pressure differences was poor on occasions. For example, in nine patients (Table 3) brain tissue pressure was close to or more than 2SD below ventricular readings.

**Table 3:** Differences in pressure between brain sites (tissue minus ventricle) in 17 patients studied during the course of 5 days of multiparameter neuromonitoring

Differences in pressure between brain sites (tissue minus ventricle, mmHg)					
ID	Number	Range		Mean	SD
1	712	−4.92	7.17	0.0131	1.77349
2	717	−29.24	12.73	1.4705	3.16501
3	703	−9.11	10.81	−1.2595	1.96992
4	687	−8.82	16.06	8.0829	2.55573
5	697	−6.16	7.27	−0.9382	1.62618
6	629	−4.75	12.38	−0.2310	1.53359
7	556	−5.99	6.52	−3.0780	2.71394
8	620	−9.65	6.28	0.4208	2.34739
9	715	−11.30	9.27	−4.9198	1.94478
10	584	−13.78	9.68	−3.6312	2.28286
11	803	−29.23	15.63	−4.7711	3.54949
12	274	−11.83	8.97	−5.5260	2.09633
13	719	−15.90	3.62	−4.9006	1.82303
14	694	−5.02	2.08	−1.7453	1.11834
15	702	−2.16	19.15	4.2851	2.90398
16	172	−25.19	18.34	2.3445	5.16720
17	307	−6.40	11.74	0.8516	2.82747

Number of pressure measurement pairs, range of differences, mean difference between sites and standard deviation (SD)

As with temperature, factors that may predict differences in pressure across the brain were explored. For pressure, differences in outcome (favourable vs. unfavourable) were not predictive of a widening pressure difference, but patients who had undergone surgical decompression or craniectomy were more likely (*p* = 0.0284) to have poor agreement in pressure readings across the brain (mean difference = −4.4899, 95 % CI −8.4250 to −0.5548).

Table 3 presents the differences in paired readings for each patient with a maximum dissociation between sites of more than 10 mmHg (11 patients). In four patients (Patients 4, 6, 15 and17) maximal tissue pressure readings were 10 mmHg or more, higher than the corresponding ventricular pressure reading. In a further four patients (Patients 9, 10, 12 and13) the maximal difference was negative, tissue pressure being at least 10 mmHg lower than ventricular pressure. In three patients (Patients 2, 11 and 16) tissue pressure, at times, was more than 10 mmHg higher, (and lower) than ventricular pressure.

Of the remainder of the variables explored, SpO_2_ was not a significant predictor of regional ICP difference (*p* = 0.220). For MAP, cerebral perfusion pressure (CPP) and heart rate (*p* < 0.0001, *p* < 0.0001 and *p* < 0.0001 respectively) significant differences were found but were too small to be of clinical relevance.

## Discussion

Neuromonitoring is appealing, providing ‘real-time’ values about changes relevant to brain physiology and tissue survival which would otherwise be inaccessible to the clinician [8]. When pressure and temperature readings change outside the guidelines for care, clinical decisions need to be made. Fundamentally, clinicians must accept the manufacturer’s stated accuracy because there is neither the time nor the facility to check at the bedside. When it comes to the brain, questions about the most appropriate site to position the sensor is a valid one. For temperature and pressure, we might posit tissue or ventricle? What difference might exist between tissue and a site deep within the brain filled with fluid? It is helpful to know whether differences do exist; and if they do, whether they are clinically relevant.

In health, the notion of a temperature gradient from body core to skin surface is well recognized [12, 13], but what about the injured brain? Well insulated within the skull and receiving arterial inflow, the brain is usually considered to be ‘hotter’ than the body [14]; the polarity of difference typically being positive—in some reports as small as 0.3 °C [15], in others much larger and of the order of 2 °C in some patients and at some times [16]. More recently, however, studies have emerged to show that the injured brain is not always a ‘hotter’ organ. Furthermore, the effects of treatment can influence the brain–body temperature gradient [17]. This systematic review of the literature highlights the potential impact of therapeutic cooling and moderate hypothermia for effects on reversal of the temperature gradient polarity (brain temperature falling below body temperature). Other factors can also play a role. When a part of the skull is removed surgically, it might be expected that the rate of heat exchange from local brain structures would increase, a finding supported by Nakagawa et al. [18]. This group showed that the effect of hemicraniectomy influences brain–body temperature gradients with brain tissue relative to the core (bladder) temperature approximately 1 °C lower.

What of temperature gradients within the brain? Computer models [19] reveal that intact human brain temperature can be substantially different from deep brain values only in the regions near the brain surface. This is due to the ‘temperature shielding’ effect of cerebral blood flow [20]. In patients with closed head injury, Fountas et al. [21] report no significant difference between intraventricular and intraparenchymal readings using a ‘pull-out process’ to assess the temperature gradient at 1 cm intervals outward from the lateral ventricle. It is not possible to determine from their data what effect a breach of insulation (e.g. craniectomy) would have on brain temperature gradients.

In this study we report results from a mixed population of patients with severe TBI, with and without multiple trauma and with and without craniotomy or craniectomy. In more than 9000 paired temperature measurements (brain tissue–ventricle), differences between brain sites were clinically insignificant. The overall agreement for measurement pairs for the cohort was good, with 96 % of pairs within 2SD (−0.4 to 0.4 °C). Differences in temperature measurement pairs outside the limits of agreement were noted in two patients only; both non-survivors. In these two patients, temperature dissociation between the sites began 2 hours before death. Brain tissue temperature dropped below ventricular and rectal temperatures. These findings support our earlier observations of a fall in brain temperature of approximately 3 °C below body (rectal) temperature before death and in association with an absence of cerebral blood flow (on CT perfusion) [22]. This finding gives further support for the justification of direct measurement of the tissue of interest (brain) rather than of surrogate measures taken from other sites.

In this cohort, the majority of patients underwent craniectomy. It might therefore be expected that local cooling might occur such that parenchymal readings would be lower than values deep within the brain. This is not borne out in the study. Clinicians can be reassured that the site of the sensor within the brain does not contribute to significant reading variability and that tissue readings are as good a clinical indicator of global temperature as are ventricular readings. The caveat, however, is that where two sensors might be considered clinically relevant, a negative temperature dissociation (brain tissue below ventricular temperature) of the order of 0.5 °C may predict a worsening of outcome or a potentially demonstrable impact of therapeutic cooling. Currently, there is little work in this area.

A similarly large number of ICP reading pairs were also obtained during the course of 5 days of monitoring. We know from our own previous bench-testing and bedside calibration tests, using the same sensors as in the current study, that the stability and accuracy of the combined temperature and pressure sensors we have used [3, 23] provide reliable and accurate readings. We have also demonstrated the performance of the sensors, positioned in tissue and ventricle and at varying times after explantation [23]. Differences between sensor and test pressures are clinically tolerable to give good measurement performance [23]. For further information on sensor depth and site, refer to [23].

In this ‘real-world’ setting of neurological monitoring within a surgical ICU there was an almost equivalent division of paired mean pressure differences around the mean (93 % of ICP tissue–ventricle pairs fall within 2SD). However, agreement was poor, at times, in some patients. Brain tissue pressure dissociated from ventricular pressure (above or below it) by more than 10 mmHg; tissue pressure underestimating as well as overestimating ventricular pressure. Review of the data for pressure dissociations alongside our clinical notes offers three ‘candidate’ events worthy of future investigation: tracheal suction, transport of patients (e.g. return from CT scan), and recording pressure values immediately after probe insertion (i.e. at the start of the patient’s neurological monitoring). For the most part, however, it was not possible to offer precise explanations for pressure dissociation between tissue and ventricular sites but persistence of pressure dissociation >10 mmHg was not noted.

Pressure reading dissociation between intracerebral sites was observed by Gambardella et al. [24] in a brain tissue pressure validation study. In the current study, transient and significant gradients in pressure of the order of 10–20 mmHg were reported between two sensors when positioned bilaterally in a small number of patients with unilateral mass lesions.

Controversy continues over the question of compartmental pressure differences owing to supratentorial mass lesions [6, 24]. It would be reasonable, in patients with a supratentorial, unilateral lesion, to expect a difference in tissue pressure at the site of the mass compared with the contralateral tissue pressure, but what do we know of differences between tissue and the ventricle with or without mass lesions, or even the effect of tissue decompression on differences between tissue or ventricular pressure readings. This is a clinically relevant issue because, as in our series, many patients admitted for neurosurgical management undergo hemi-or bilateral craniectomy. With the exception of a small number of readings in some patients and at some times, agreement between pressure values at two intracerebral sites were, on balance, within 10 mmHg in either direction of the mean despite major surgery and removal of a bone flap. Intraparenchymal pressure and temperature monitoring provides acceptable information to guide and direct clinical management of brain-injured patients in the neurosurgical ICU. We do, however, acknowledge the limitations of the study. Whilst a large number of paired readings were obtained, the study sample remains relatively small. However, this rather reflects the relatively small population of the host country for this study compared with previous studies [25], the consequence of which is the duration of time required for recruitment of a larger patient series. We also recognize the need for a further prospective study to establish the nature of the large dissociation (>10 mmHg) in pressure between parenchymal and ventricular readings, most importantly to rule out spurious readings, and the cause.

## Conclusions

There is good overall agreement between paired temperature measurements obtained from deep white matter and brain ventricle in patients with and without early neurosurgery. For paired pressure measurements, 93.5 % of readings were within 2SD of the mean difference. The majority of paired differences were within 10 mmHg. However, at times, clinically significant differences, greater than 10 mmHg and reaching 29 mmHg in one case, were observed in 11 of 17 patients. The periods whereby pressure readings (between tissue and ventricle) varied by more than 10 mmHg, despite being rather short episodes, could, in real time, act as a ‘trigger’ to the clinician to order additional investigation (e.g. CT scan, change of drug therapy) which might be unnecessary or, in the extreme, result in emergency surgery; hemicraniectomy, for example, which might in fact be unwarranted or even deleterious to patient outcome.

## Key messages

- There is good overall agreement between paired temperature measurements obtained from deep white matter and lateral ventricle in patients with and without early neurosurgery.
- The sensor site does not significantly contribute to regional variations in brain temperature.
- Although the majority of tissue–ventricular pressure readings are within 10 mmHg, clinically relevant differences (>10 mmHg) occurred, often as non-specific pressure deviation episodes, in this mixed cohort of TBI patients.
- Further work is warranted to establish the clinical events linked to tissue–ventricular pressure dissociations.

## Abbreviations

AIS: Abbreviated Injury Scale
CO_2_: Carbon dioxide
CPP: Cerebral perfusion pressure
CSF: Cerebrospinal fluid
CT: Computed tomography
GCS: Glasgow Coma Scale
GOS: Glasgow Outcome Score
ICIP: Intellivue Clinical Information Portfolio
ICP: Intracranial pressure
ISS: Injury Severity Score
MAP: Mean arterial pressure
Pb_ti_O_2_: Brain tissue oxygen partial pressure
SD: Standard deviation
SpO_2_: Peripheral capillary oxygen saturation
TBI: Traumatic brain injury
